# Sleep Hygiene Practices and Its Impact on Mental Health and Functional Performance Among Adults in Tabuk City: A Cross-Sectional Study

**DOI:** 10.7759/cureus.36221

**Published:** 2023-03-16

**Authors:** Eman M Alanazi, Abeer Mohammed M Alanazi, Asmaa Hamed Albuhairy, Alshaymaa Akram A Alanazi

**Affiliations:** 1 Department of Family Medicine, King Fahad Specialist Hospital, Tabuk, SAU; 2 Department of Pediatrics, Faculty of Medicine, University of Tabuk, Tabuk, SAU; 3 Department of Family Medicine, Faculty of Medicine, University of Tabuk, Tabuk, SAU

**Keywords:** mental health, depression, daytime sleepiness, sleep quality, insomnia, sleep hygiene

## Abstract

Background

Poor or imperfect sleep hygiene practices include all factors that promote arousal or disrupt the normal balance of the sleep-wake cycle. It is necessary to clarify the relationship between sleep hygiene behaviors and a person’s mental health. This may allow a better understanding of this problem and might help design effective awareness programs about good sleep hygiene practices for reducing the serious outcomes of this problem. Therefore, the current study was conducted to evaluate sleep hygiene practices and assess the impact of sleep hygiene on sleep quality and the mental health of the adult population of Tabuk city, Saudi Arabia.

Methodology

This cross-sectional, survey-based study was conducted in Tabuk city, Saudi Arabia in 2022. All adult residents of Tabuk city, Saudi Arabia were invited to participate. Participants with incomplete data were excluded from the study. A self-administered questionnaire was developed by the researchers to assess sleep hygiene practices and their impact on the sleep quality and mental health of the study participants.

Results

The study included 384 adults. There was a significant association between the frequency of sleep problems and poor sleep hygiene practices (p < 0.001). The percentage of subjects who had problems sleeping during the past three months was significantly higher among those having poor sleep hygiene practices (76.5%) than their counterparts (56.1%). The rates of excessive or severe daytime sleepiness were significantly higher among individuals with poor hygiene practices (22.5% versus 11.7% and 5.2% versus 1.2%, p = 0.001). Participants with depression were found to be significantly higher among the poor hygiene group (75.8%) in comparison to those having good hygiene practices (59.6%) (p = 0.001).

Conclusions

The findings of the present study indicate significant associations between poor sleep hygiene practices and sleep problems, daytime sleepiness, and depression among adult residents of Tabuk city, Saudi Arabia.

## Introduction

Sleep is necessary for preserving both the physical and mental health of humans. Additionally, it is essential for maintaining cognitive capabilities such as memory, learning, and the capacity to perform complex mental tasks [1].

Sleep hygiene is a growing public health concern globally as well as in Saudi Arabia [2]. Sleep hygiene is the set of behavioral and environmental aspects that support healthy sleep patterns [3].

Poor or imperfect sleep hygiene practices include all factors that promote arousal or disrupt the normal balance of the sleep-wake cycle. These include various factors involving inconsistent sleep schedules and regular usage of stimulants, especially before bedtime [4].

Previous research has indicated that poor sleep hygiene practices have a significant impact on sleep quality and duration. Insomnia may have a detrimental effect on a person’s well-being, and poor sleep patterns have been linked to a range of mental and physical disorders [5].

These findings emphasize the importance of evaluating sleep hygiene practices in the general population to clarify their relationship to the individual’s mental well-being and functional performance [6]. This may allow a better understanding of this problem and might help design effective awareness programs about good sleep hygiene practices for reducing the serious outcomes of this problem [7].

Therefore, this study was conducted to evaluate sleep hygiene practices and assess the impact of sleep hygiene on the sleep quality and mental health of the adult population of Tabuk city, Saudi Arabia.

## Materials and methods

Study design, date, and setting

This cross-sectional, survey-based study was conducted in Tabuk city, Saudi Arabia in 2022.

Sample size and sampling technique

The sample size was estimated using an online sample size calculator (Raosoft, http://www.raosoft.com/samplesize.html) with a margin of error of 5% and a confidence interval of 95%, assuming an average response for most questions of 50%, and depending on an average population size of 372,021 adult population in Tabuk city. The required sample size was 384. The participants were recruited by a convenient sampling method.

Inclusion and exclusion criteria

All adult residents of Tabuk city, Saudi Arabia were invited to participate. Participants with incomplete data were excluded from the study.

Data collection tool

A self-administered questionnaire was developed by the researchers to assess sleep hygiene practices and their impact on the mental health of the study participants. This tool included five sections.

Section A assessed the sociodemographic data of the participants, including age, weight, height, level of education, marital status, employment, residence, and nationality.

Section B was designed to measure sleep hygiene practices among the subjects. The sleep hygiene items were based on an earlier study [8]. The instruction for the sleep hygiene items was “Below is a list of behaviors and circumstances that captures what people might do during the day, evening, or night. Please report how much you have engaged in these behaviors and circumstances during the past month by indicating how much you agree with the statements from 1 (strongly disagree) to 5 (strongly agree).” The nine sleep hygiene items were “I have been taking naps during the day,” “I have had an irregular sleep schedule, i.e., using differing set times for going to bed and getting up from bed,” “I have been drinking alcohol late in the evening,” “I have been using nicotine late in the evening,” “I have been drinking caffeinated drinks late in the evening,” “I have gone to bed hungry or too full or I have been drinking liquids late in the evening,” “I have been exercising late in the evening,” “I have been disturbed by light or noise while in bed,” and “I have had an uncomfortable sleep environment in my bedroom, e.g., uncomfortable bed or temperature.” Further, the total sleep hygiene practices score was calculated by the sum of the recorded scores for each item. The calculated total sleep hygiene score ranged from 9.0 to 44.0, with a median score of 25.0 (IQR = 22.0-28.0) Participants who had a total sleep hygiene score of 25.0 or more were considered to have poor sleep hygiene practices.

Section C assessed the presence of sleep problems and the quantity of these problems. It started with the question “have you had problems sleeping during the past three months?” and its answers were either “yes” or “no,” followed by the five-point scale of the Basic Nordic Sleep Questionnaire (BNSQ) [9] that stresses on how many nights/days per week something happens. The basic scale is 1, “never or less than once per month”; 2, “less than once per week”; 3, “on 1-2 nights per week”; 4, “on 3-5 nights per week”; and 5, “every night or almost every night.” Furthermore, there were two questions to assess “how many minutes are you awake before you fall asleep?” and “if you wake up at night, how many minutes are you awake?.”

Section D assessed the sleepiness criteria according to the Epworth Sleepiness Scale (ESS) [10], which represents daytime sleepiness and consists of eight items rated on a four-point scale. The instruction was “how likely are you to doze off or fall asleep in the following situations, in comparison to feeling just tired? Use the following scale to choose the most appropriate number for each situation (0 = would never doze; 1 = slight chance of dozing; 2 = moderate chance of dozing; and 3 = high chance of dozing).” The total ESS score was calculated and graded as follows: ESS <10 corresponds to the absence of sleepiness, ESS 11-15 suggests excessive daytime sleepiness, and ESS >16 indicates severe sleepiness [10].

Section E used the 10-item version of the Center for Epidemiologic Studies Short Depression Scale (CES-D-10), which is a reliable tool for measuring depression [11]. The CES-D-10 assesses depressive symptoms in the past week. It includes three items on depressed affect, five items on somatic symptoms, and two on positive affect. Options for each item range from “rarely or none of the time” (score of 0) to “all of the time” (score of 3). Scoring is reversed for items five and eight, which are positive affect statements. The total score can range from 0 to 30. Higher scores suggest a greater severity of symptoms. The optimal cutoff value of the CES-D-10 scale was ≥10 according to Fu et al. [12]. Subjects with a score above the cutoff value were classified as having depression.

Ethical considerations

The study obtained ethical approval from the Research Ethics Committee of the Directorate of Health Affairs in Tabuk city, Saudi Arabia (TU-077/022/119). Participants were informed about the study objectives, methodology, risks, and benefits. Subjects who agreed to fill out the questionnaire imply that they agreed to participate in the study. The participants’ confidentiality was preserved, and the data will not be used for any other purpose outside this study.

Statistical analysis

Data were tabulated and analyzed using the statistical package SPSS version 22 (IBM Corp., Armonk, NY, USA). Categorical variables were summarized as frequencies and percentages, and the associations between variables were tested using the chi-square tests (Pearson’s chi-square for independence or Fisher exact tests as appropriate). Continuous data were tested for normality using the Shapiro-Wilk test. Normally distributed data were displayed as mean ± SD. The skewed data were represented as the median and IQR (25th-75th percentiles) and were compared using the non-parametric Mann-Whitney U test. A p-value of <0.05 was considered statistically significant.

## Results

**Figure 1:**
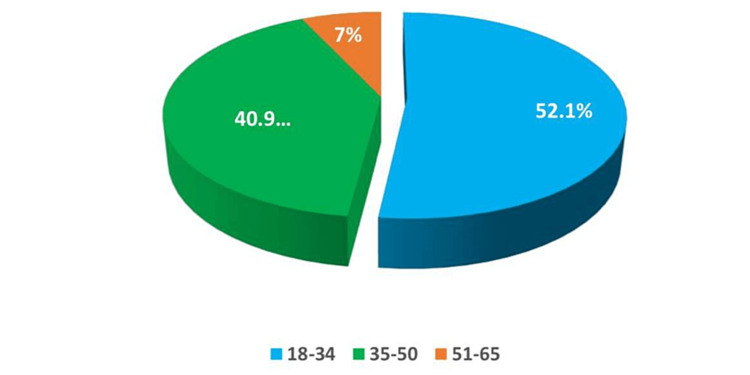
Distribution of the age groups of the study participants.

**Figure 2:**
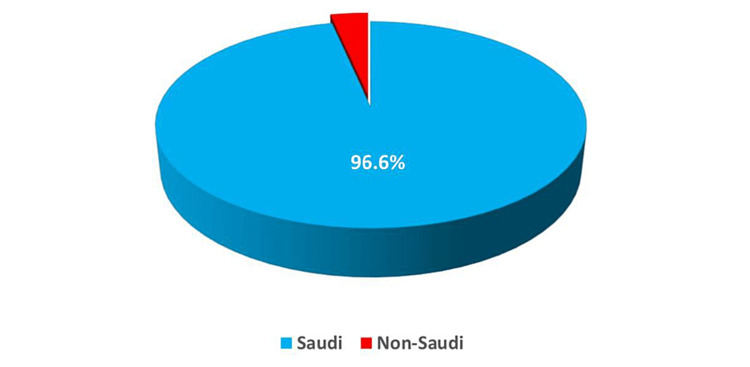
Distribution of the nationality of the study participants.

**Figure 3:**
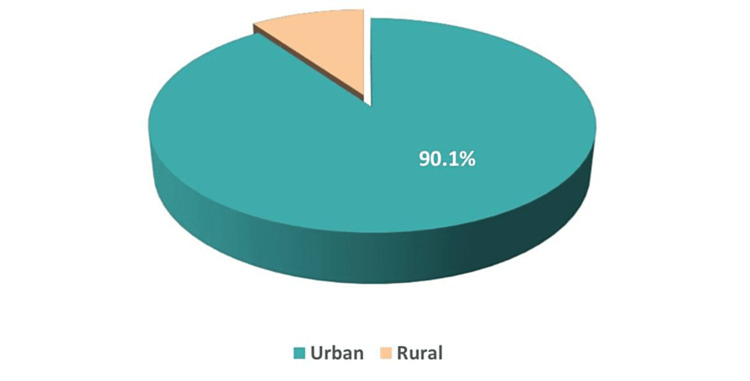
Distribution of the residence of the study participants.

This survey-based study incorporated 384 adult residents of Tabuk city, Saudi Arabia. The most frequently participating age groups were 18-34 and 35-50 years (52.1% and 40.9%, respectively) (Figure 1). Most participants were Saudi (96.6%) and urban (90.1%) (Figures 2, 3). Furthermore, Table 1 shows that most had a university education (73.4%), and the marital status varied, with 51.3% married, while the single, widowed, and divorced subjects constituted 41.7%, 4.4%, and 2.6%, respectively. The greatest percentages were employed (41.7%) and students (33.1%). Their mean body mass index (BMI) was 27.23 ± 6.39 kg/m^2^.

**Table 1:** Sociodemographic characteristics of the study participants (N = 384). BMI: body mass index

		N	%
Level of education	University	282	73.4
	Preuniversity	68	17.7
	Postgraduate	19	4.9
	Read and write	15	3.9
Marital status	Married	197	51.3
	Single	160	41.7
	Widow	17	4.4
	Divorced	10	2.6
Occupation	Employed	160	41.7
	Student	127	33.1
	Non-employed	75	19.5
	Sick leave or pension	22	5.7%
BMI, kg/m^2^	Minimum-Maximum	14.57-68.49	
	Mean ± SD	27.23 ± 6.39	

The participants’ responses to sleep hygiene practices items are shown in Table 2. The calculated total sleep hygiene score ranged from 9.0 to 44.0, with a median score of 25.0 (IQR = 22.0-28.0) and a mean score of 24.9 ± 5.3. Participants who had a total sleep hygiene score of 25.0 or more were considered to have poor sleep hygiene practices (N = 213, 55.5%), while a score less than 25 reflected good sleep hygiene practices (N = 171, 44.5%).

**Table 2:** Sleep hygiene practices among the study participants (N = 384).

		N	%
I have been taking naps during the day	Strongly disagree	35	9.1
	Disagree	36	9.4
	Neutral	71	18.5
	Agree	155	40.4
	Strongly agree	87	22.7
I have had an irregular sleep schedule	Strongly disagree	38	9.9
	Disagree	83	21.6
	Neutral	49	12.8
	Agree	149	38.8
	Strongly agree	65	16.9
I have been drinking alcohol late in the evening	Strongly disagree	57	14.8
	Disagree	88	22.9
	Neutral	59	15.4
	Agree	129	33.6
	Strongly agree	51	13.3
I have been using nicotine late in the evening	Strongly disagree	279	72.7
	Disagree	39	10.2
	Neutral	20	5.2
	Agree	32	8.3
	Strongly agree	14	3.6
I have been drinking caffeinated drinks late in the evening	Strongly disagree	269	70.1
	Disagree	53	13.8
	Neutral	22	5.7
	Agree	23	6.0
	Strongly agree	17	4.4
I have gone to bed hungry or too full, or I have been drinking liquids late in the evening	Strongly disagree	69	18.0
	Disagree	58	15.1
	Neutral	82	21.4
	Agree	135	35.2
	Strongly agree	40	10.4
I have been exercising late in the evening	Strongly disagree	100	26.0
	Disagree	132	34.4
	Neutral	75	19.5
	Agree	64	16.7
	Strongly agree	13	3.4
I have been disturbed by light or noise while in bed	Strongly disagree	30	7.8
	Disagree	51	13.3
	Neutral	41	10.7
	Agree	128	33.3
	Strongly agree	134	34.9
I have had an uncomfortable sleep environment in my bedroom	Strongly disagree	93	24.2
	Disagree	114	29.7
	Neutral	59	15.4
	Agree	91	23.7
	Strongly agree	27	7.0

There was a significant association between the frequency of sleep problems and poor sleep hygiene practices (p < 0.001). The percentage of subjects who had problems sleeping during the past three months was significantly higher among those having poor sleep hygiene practices (76.5%) than their counterparts (56.1%). Moreover, the magnitude of sleep problems was significantly associated with sleep hygiene practices (p = 0.001). The percentages of respondents who experienced everyday sleeping problems were significantly higher among the poor sleep hygiene practices group (14.1%) than among those with good practices (7%). Furthermore, the medians of the number of minutes the participants were awake before falling asleep or remaining awake if woke up at night were significantly different (p < 0.05) (Table 3).

**Table 3:** Associations between sleep hygiene practices and the frequency and magnitude of sleep problems. IQR: interquartile range *: Significant at p < 0.05.

		Sleep hygiene practices		P-Vvalue
		Good, N = 171 (44.5%)	Poor, N = 213 (55.5%)	
Have you had problems sleeping during the past three months?	No	75	50	<0.001*
		43.9%	23.5%	
	Yes	96	163	
		56.1%	76.5%	
How often have you experienced problems sleeping during the past three months?	Never or less than once per month	66	54	0.001*
		38.6%	25.4%	
	One or two days per week	52	64	
		30.4%	30.0%	
	More than two days per week	41	65	
		24.0%	30.5%	
	Every day	12	30	
		7.0%	14.1%	
On average, how many minutes are you awake before you fall asleep?	Minimum	1.0	2.0	<0.001*
	Maximum	120.0	180.0	
	Median	20.0	30.0	
	IQR	10.0–30.0	15.0–45.0	
On average, if you wake up at night, how many minutes are you awake?	Minimum	0.0	0.0	0.010*
	Maximum	120.0	120.0	
	Median	10.0	11.0	
	IQR	5.0–20.0	5.0–30.0	

**Figure 4:**
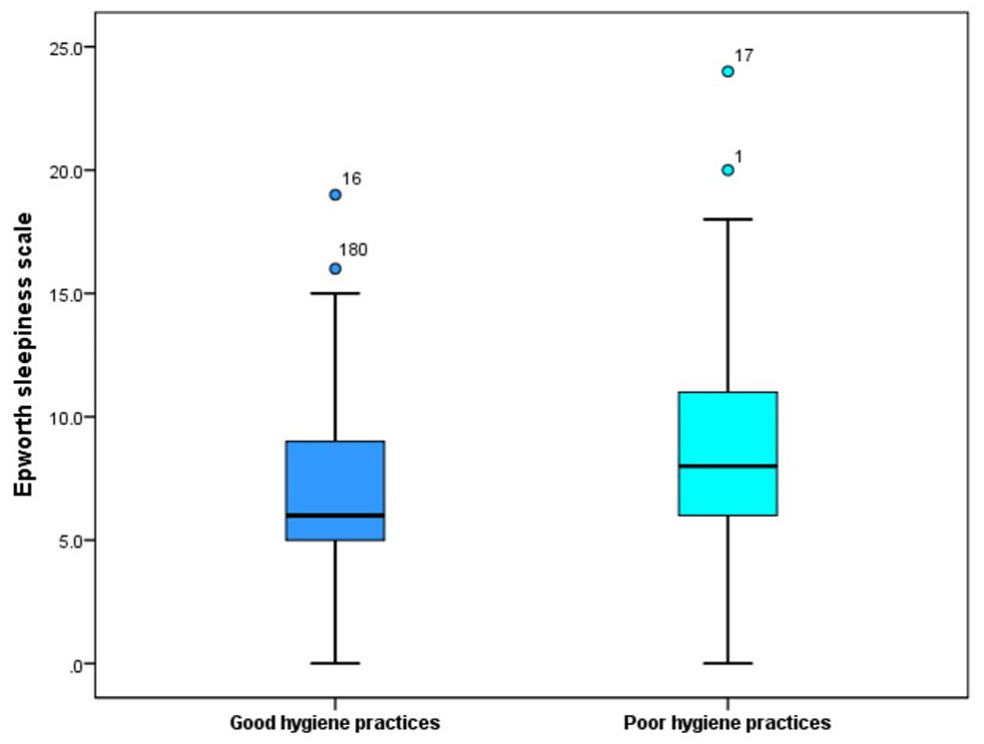
Comparison of Epworth Sleepiness Scale between subjects with good or poor sleep hygiene practices.

**Figure 5:**
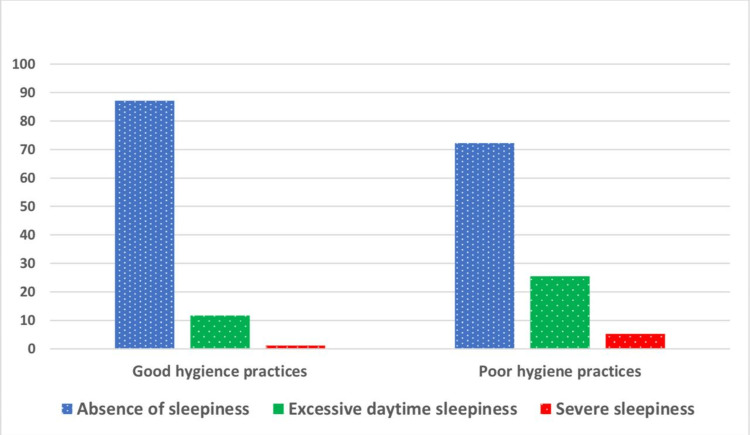
Association between sleep hygiene practices and daytime sleepiness.

The calculated minimum ESS was 0.0 and the maximum was 24.0, with a median of 8.0 (IQR = 5.0-10.0). According to the ESS, the participating subjects were graded as the absence of sleepiness (78.9%), excessive daytime sleepiness (17.7%), or severe sleepiness (3.4%). Figure 4 shows a significantly higher median ESS score in the poor hygiene group (8.0) than in the good hygiene group (6.0) (p < 0.001). The rates of excessive or severe daytime sleepiness were significantly higher among individuals with poor hygiene practices (22.5% versus 11.7% and 5.2% versus 1.2%, p = 0.001), as illustrated in Figure 5.

**Figure 6:**
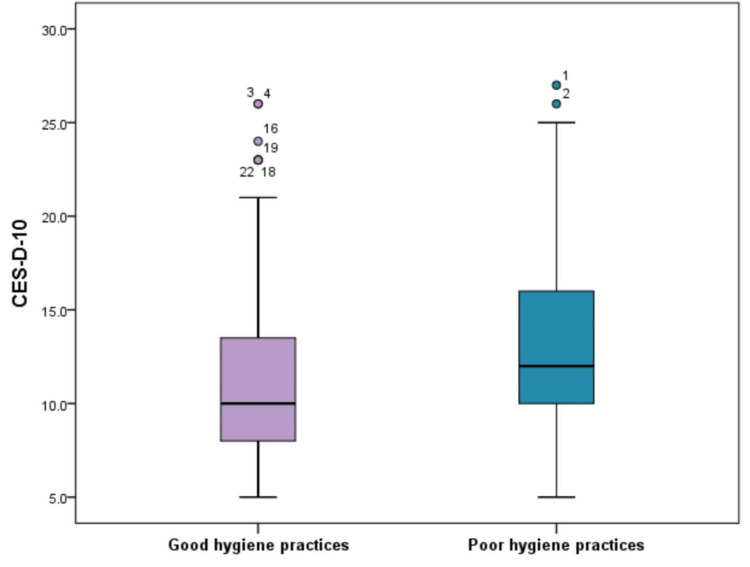
Comparison of CES-D-10 screening score for depression between subjects with good or poor sleep hygiene practices. CES-D-10: 10-item version of the Center for Epidemiologic Studies Short Depression Scale

**Figure 7:**
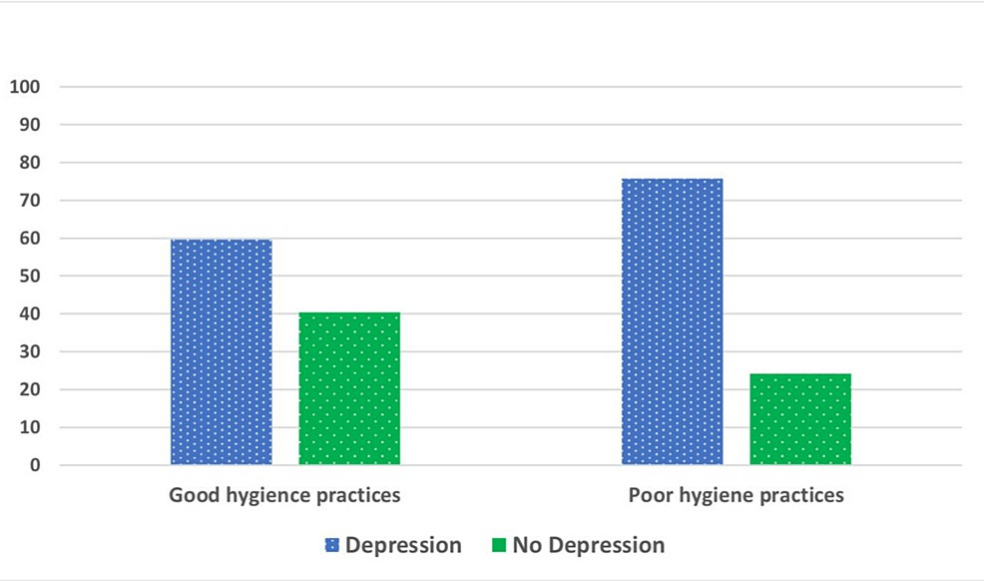
Association between participant’s mental health and sleep hygiene practices.

We calculated the total CES-D-10 screening score for depression. It ranged from 5.0 to 27.0, with a median of 11.0 (IQR = 9.0-15.0), and a mean score of 12.5 ± 4.9. Based on the recommended cutoff value of ≥10, the subjects were classified as either having depression (68.6%) or not (31.4%). Figure 6 shows a significantly higher median CES-D-10 score among the poor hygiene group than the good hygiene group (12.0 versus 10.0, p < 0.001). Further, participants with depression were found to be significantly higher among the poor hygiene group (75.8%) in comparison to those having good hygiene practices (59.6%) (p = 0.001) (Figure 7).

## Discussion

Sleep problems including poor sleep, insomnia, and daytime sleepiness are regarded as important public health issues due to their growing frequency and potentially dangerous effects. These effects include physical and mental health impairments, decreased productivity, higher risk of accidents, greater use of medical services, and increased risk of psychiatric illnesses [13].

Inadequate sleep hygiene has been considered one of the several factors contributing to poor sleep and insomnia. This survey-based study aimed to investigate the relationship between sleep hygiene practices and sleep problems and the mental health of the adult population living in Tabuk city, Saudi Arabia.

In this study, 55.5% of the participants reported poor sleep hygiene practices, and there were significant associations between poor sleep hygiene and the prevalence of sleep problems. Moreover, respondents who experienced everyday sleeping problems were significantly higher among poor sleepers (14.1%) than good sleepers (7%). In this concern, an earlier population-based study found that inadequate sleep hygiene practices such as smoking, drinking alcohol, napping, and sleeping on weekends were significantly higher among subjects with insomnia compared to the age- and sex-matched control group of good sleepers [14]. Gellis et al. [15] also reported significant relationships between inconsistent sleep schedules and behaviors that encourage arousal near bedtime and insomnia severity in college students. A more recent study reported significant associations between good sleep hygiene in the form of sleeping in a comfortable environment, limiting naps to 30 minutes, maintaining a consistent wake time, and reporting better sleep quality/efficiency in a sample of young adults [16]. Furthermore, Carrión-Pantoja et al. [17] reported significant associations between sleep hygiene, insomnia symptoms, and depression, which all were correlated with the academic performance of university students. Therefore, the involvement of sleep hygiene in multicomponent intervention therapies to attain efficacious therapies for insomnia is highly warranted.

This survey explored higher rates of excessive or severe daytime sleepiness among individuals with poor hygiene practices (22.5% versus 11.7% and 5.2% versus 1.2%, p = 0.001). This finding agrees with earlier studies [6,18] reporting poor sleep hygiene as one of the contributing factors for both insomnia and excessive daytime sleepiness. A corresponding survey in southwest Saudi Arabia also showed worse sleep hygiene practices among participants suffering from insomnia symptoms and excessive daytime sleepiness [19]. Another study found that irregular and unhygienic sleep practices result in sleep deficiency and daytime sleepiness [20]. It is worth mentioning that daytime sleepiness affects neuropsychiatric diseases, increases the risk of harm at work or home, contributes to car accidents, and reduces overall productivity [21]. A total of 963 college students showed a significantly increased risk of chronic mental disorders among subjects with excessive daytime sleepiness (odds ratio (OR) = 3.65; 95% confidence interval (CI) = 2.56-4.91) and poor sleep quality (OR = 4.76; 95% CI = 3.11-7.29) [22]. Sleep hygiene is considered a modifiable behavior that can be targeted to improve individuals’ well-being. Hence, intervention programs for adjusting sleep hygiene practices are crucial to minimizing the risk of sleep problems and their related disorders [23].

Worsening sleep hygiene may predispose to psychiatric disorders such as depression [24]. Hence, this study investigated the relationship between sleep hygiene and the prevalence of depression. There was a significantly higher rate of depression among persons with poor sleep hygiene practices (75.8%). This coincides with Çelik et al. [25] who found 3.28 times increased risk of depressive symptoms in students with poor sleep quality. Similarly, Gupta et al. [24] highlighted significant associations between depression among adolescents and poorer sleep quality, longer sleep onset latency, and shorter sleep duration. Many longitudinal studies have revealed that sleep disturbance is not only a prodromal symptom of depression but also an independent risk factor for subsequent depression, and negatively affects the course of the disease with increased suicide rates [26].

Moreover, sleep quality was significantly linked to the severity of depression [24]. A recent study reported that marked daily irregularity in sleep patterns among 100 undergraduate and graduate young adults significantly predicted higher levels of severity of depression after controlling for demographic and clinical characteristics [27].

The relationship between sleep disturbance and depression might be explained in light of the inflammation hypothesis which highlights an association between poor sleep quality, increased levels of inflammatory cytokines such as interleukin-6 and C-reactive protein, and depression. However, the exact interaction between them remains unclear. Additionally, it has been suggested that sleep disorders are associated with disturbances in the circadian rhythm which has also been observed in patients with major depressive disorders [28]. Education and the application of policies regarding sleep hygiene may prevent, in some cases, the development of depression [29].

Limitations

There are some limitations that must be noted when interpreting the findings of this study. First, being cross-sectional makes it difficult to recognize the direction of a particular association and advocate causation. Furthermore, the subjective measurements of variables may increase the likelihood of biases. However, the application of valid data collection scales may reduce this risk.

## Conclusions

The findings of the present study indicate significant associations between poor sleep hygiene practices and sleep problems, daytime sleepiness, and depression among adult residents of Tabuk city, Saudi Arabia. These findings shed light on the necessity of designing effective awareness and intervention programs aiming at the development of sleep hygiene practices and the improvement of sleep quality among the Saudi population. These measures will unquestionably improve physiological performance and psychological well-being and bring a better quality of life. Sleep hygiene education must be a part of broader primary prevention strategies for psychiatric disorders. Further, prospective cohort and intervention studies are recommended to investigate the possible contributing role of poor sleep hygiene in the development of insomnia and depression.
